# Vaccine-elicited systemic and mucosal humoral responses of lactating rhesus monkeys vaccinated with the transmitted/founder HIV Envelope 1086C

**DOI:** 10.1186/1742-4690-9-S2-O20

**Published:** 2012-09-13

**Authors:** G Fouda, J Amos, AB Wilks, A Chand, D Montefiori, B Haynes, N Letvin, D Pickup, H Liao, SR Permar

**Affiliations:** 1 Beth Israel Deaconess Medical Center, Boston, MA, USA; 2 Duke University Medical Center, Durham, NC, USA

## Background

We previously demonstrated that vaccination of lactating rhesus monkeys with a DNA prime/ vector boost strategy induces strong SIV-specific cellular immune responses, but limited Envelope-specific humoral responses in breast milk. Therefore, we sought to improve vaccine-elicited Envelope-specific antibody responses in the milk compartment by using a transmitted/founder (T/F) HIV Envelope immunogen in a prime-boost strategy modeled after that of the moderately-successful RV144 HIV vaccine trial.

## Methods

Eight female, hormone-induced lactating rhesus monkeys were intramuscularly primed with either recombinant DNA (n = 4) or MVA pox virus vector (n = 4) expressing the T/F clade C HIV Envelope 1086C. All animals were intramuscularly boosted twice with the 1086C gp120 protein and the adjuvant MF59. Milk, vaginal, rectal and plasma samples were assessed for HIV Envelope-binding IgG and IgA responses. Anti-V1V2 antibodies and neutralization responses were also measured in milk and plasma.

## Results

Envelope 1086C-binding IgG responses were detected in plasma, milk, and vaginal samples of all vaccinated animals and two of four rectal samples from MVA-vaccinated animals. Moreover, anti-V1V2 IgG antibodies were detected in all plasma, but only one milk sample. Low magnitude Envelope 1086C-specific IgA responses were detected in milk of two of four DNA-primed and three of four MVA-primed animals, but in none of the rectal samples. In contrast, all vaginal samples from MVA-primed, but none from DNA-primed, animals had detectable Envelope-specific IgA. Remarkably, strong tier 1 and low to moderate tier 2 neutralization was detected in plasma and milk of each group. The plasma neutralization titers against MW965 (clade C tier 1, p=0.03) and CAP45 (clade C tier 2, p=0.03) were significantly higher in MVA-primed than DNA-primed animals.

## Conclusion

MVA prime/ T/F Envelope protein boost strategy appears to induce stronger systemic and mucosal binding and neutralizing antibody responses than the DNA prime/protein boost regimen in lactating rhesus monkeys.

